# A transport model for the permeation of colloidal silica

**DOI:** 10.1007/s11440-026-03117-z

**Published:** 2026-06-13

**Authors:** Matteo Pedrotti, Joseph Lyons, Grainne El Mountassir, Oliver Kuras, Rebecca J. Lunn

**Affiliations:** 1 Department of Civil and Environmental Engineering, University of Strathclyde, James Weir Building-Level 5, 75 Montrose Street, Glasgow, G1 1XJ Scotland, UK; 2 Natural Power, Glasgow, United Kingdom; 3 British Geological Survey, Nottinghamshire, England

**Keywords:** Colloidal silica, Electric resistivity, Gel time, Grout, Hydraulic barriers, Permeation

## Abstract

Colloidal silica grout is a relatively new grouting material and as such, it does not fit any current design standard and is missing the empirical knowledge that accompanies conventional grouting materials. Industry uptake seems slowed by (i) the lack of a grout penetration model to inform engineering design, (ii) the lack of laboratory or field data to understand grout penetration in the natural environment, specifically where there are heterogeneous soils or in the presence of in situ cations and (iii) the lack of in-situ validation methods that can demonstrate grout barrier integrity at a commercial scale. This paper presents a transport model to predict the spatio-temporal evolution of in situ colloidal silica grout permeation, accounting for the interaction between the grout and the porewater chemistry during injection. The newly developed viscosity relationship is implemented within a flow and transport model to predict grout permeation. The model’s predictive accuracy is validated against a series of metre-scale, tank-based experiments involving varying soil permeabilities and saline groundwater conditions. Furthermore, this research evaluates the efficacy of Electrical Resistivity Tomography (ERT) as a commercial-scale monitoring tool. Results demonstrate that differential ERT analysis (comparing pre- and post-injection states) successfully delineates the grouted soil volume and barrier integrity. The findings provide a dual-track solution for geotechnical engineering: a numerical framework for design optimisation and a geophysical methodology for field-scale validation.

## Introduction

According to the International Energy Agency approximately 200 nuclear reactors are due to shut-down by 2040 [34] resulting in a significant legacy of sites for decommissioning. Decommissioning of nuclear sites presents technical challenges that are not common in traditional ground and civil engineering operations. Many nuclear facilities have operated well beyond their original design lives and are commonly located in coastal regions where environmental conditions are harsh. As a result, above- and below-ground structures may be in an advanced state of deterioration and ground contamination can originate from several sources including containment silos, storage ponds, lined or unlined legacy trenches, or accidental historic spills. In addition, decommissioning operations risk further contamination through the introduction of heavy machinery required for waste retrieval; which when brought into, or adjacent to, fragile structures can cause differential settlement resulting in increased leakage rates from containment structures [65, 92].

Subsurface hydraulic barriers are a common method within the civil engineering industry to mitigate against transport of harmful contaminants in groundwater from industrial sites [1, 2] or to create foundation treatment cut-off systems or rock-mass grouting [69, 86–88]. Traditionally, barriers are created through exhumation of a trench to the depth of the shallowest impermeable layer. This is then filled with compacted clays [43, 52, 74, 75, 94, 98], geosynthetic clays [14, 57, 60] or bentonite enhanced sand mixtures [11, 24, 29, 32, 59, 84, 96]. However, such methods are problematic on nuclear sites where the exhumed soils may be contaminated with radionuclides that are hazardous to the workforce and where operations are often very congested.

Colloidal silica (hereinafter referred to as CS) is a low viscosity, injectable alternative for the creation of near-surface, low-permeability ground barriers, without the requirement for ground excavation. Colloidal silica is injected as a suspension of silica particles with a viscosity close to that of water and a particle size of 15–30 nm. Once injected, it forms a low-permeability hydrogel within the soil pores. Formation of the hydrogel can be triggered by many different methods, including temperature increase, increased electrolyte concentration and changes to pH [42]. The gel formation time can be accurately controlled.

Colloidal silica has been investigated for a number of applications, these include: enhanced oil recovery [18, 44, 105]; boreholes sealing [66, 104]; the prevention of water ingress into tunnels [3, 4, 15, 16]; the prevention of soil liquefaction upon cyclic loading [5, 20, 21, 23, 30, 31, 36, 38, 46, 58, 76, 78, 85]; for the stabilisation of sandy soils [28, 40, 53, 64, 80–82, 95]; and soil-pile frictional interaction [10]. In the late 90 s, Lawrence Berkeley National Laboratory research group investigated the use of two hydrogels (colloidal silica hydrogel and polysiloxane crosslinked hydrogel) as waste containment technologies for active sites [26, 27, 35, 61–63, 72, 73]. More recently, colloidal silica has been proposed for near surface injections for active waste containment and encapsulation [13, 17, 25, 47, 49, 55, 56, 67, 68, 70, 71, 93, 103]. However, despite a small number of successful industrial applications [9, 13, 17, 22, 49, 100, 101, 103] industry uptake remains limited.

Current adoption is hindered by:

- The absence of robust penetration models to inform predictive design.
- Insufficient data regarding grout behaviour in heterogeneous natural environments, particularly concerning interactions with in situ porewater chemistry.
- A lack of non-destructive validation methods to confirm barrier integrity at a commercial scale.

This paper’s aim is to present a transport model able to predict in situ colloidal silica permeation, specifically accounting for the chemical interactions between the grout front and porewater cations. Model simulations and tank-based laboratory experiments are presented for a range of scenarios, varying the soil permeability and the cation content of the in-situ groundwater. Finally, given that estimation of post-injection grout position is considered one of the main factor of the overall effectiveness of any subsurface injection [19, 97, 99], Electric Resistivity Tomography (ERT) is proposed as a viable method for determination of in-situ grout barrier integrity in the field [39, 79].

## Grout modelling methodology

### Background

**Fig. 1:**
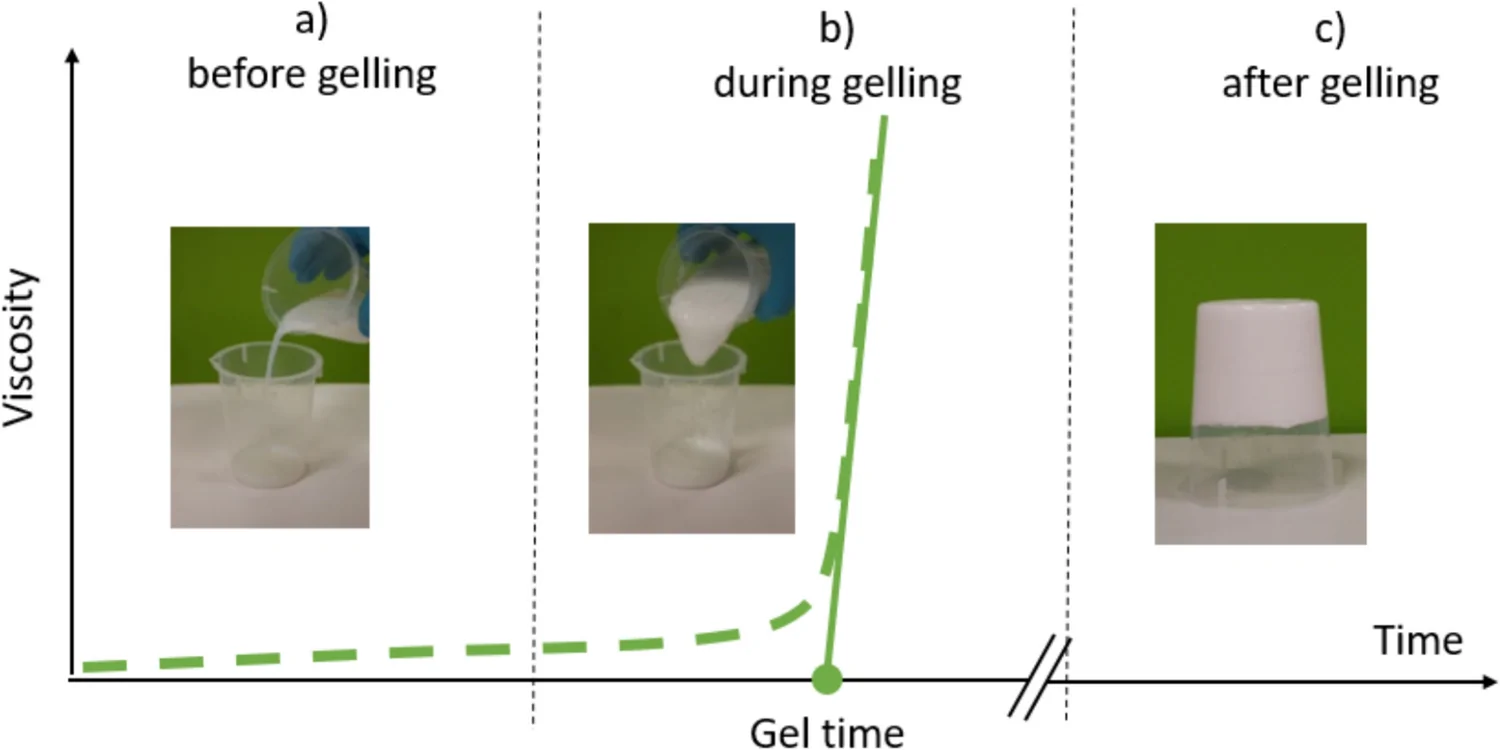
Viscosity evolution in colloidal silica during gelling (modified after Wong, Pedrotti [95])

Colloidal silica is typically induced to gel by mixing it with a solution containing cations, prior to grout injection (Fig. 1). A key control on the gel time, is the concentration and valency of the cations present in solution. As a consequence, the presence of in-situ cations such as those found in heavily contaminated sites or coastal areas, could cause premature gelling of the grout and make grout emplacement hard to control [50]. In order to obviate this problem, a pre-flush is traditionally used [12, 26, 45, 73, 89–91]. However on a legacy nuclear site, pre-flushing soils and potentially mobilising radionuclides, is not generally desirable. A lack of confidence around accurate grout emplacement has led to colloidal silica being injected in a fashion more similar to cement grouts: i.e. with short injection times and high injection pressures [23, 36–38]. However, due to ground heave, this approach prevents its use in near surface applications such as nuclear decommissioning.

The uncertainties noted above in engineering practice are also reflected in the way that silica-based hydrogel grouts are modelled. The pioneering work published by Finsterle and al. in 1994 [26, 27] paved the way for modelling the injection of gelling materials into a porous media. Key assumptions were:

- That the hydrogel behaves as a miscible fluid
- That there is no dispersion and no cations mixing at the grout front i.e. that the subsurface has been pre-flushed.
- That the viscosity evolution of the grout can be modelled without accounting explicitly for the chemical composition, but can be estimated via interpolation of the experimental data. Hence, if the grout composition changes, a new experimental dataset is required.
- That any interaction at the grout front is in the form of grout dilution which reduces the grout viscosity according to a linear mixing rule.

These assumptions effectively minimise any interaction effects between the grout and the pore fluid. For jet grouting techniques the assumptions are valid [6], they have short injection times and high injection pressures so the time available for grout front interactions is short. However, in the case of permeation grouting, where the duration of permeation (grout transport prior to gelling) is longer, a new model is required.

Similar to what recently used when modelled bio-cementing agents grouting [51, 77], in the following sections we introduce a model of grout transport, based on solution of the groundwater flow and transport equations, that allows for grout front mixing and for the influence of cations that may be present within the in-situ groundwater on grout gelling.

### General flow and transport equations

The injection of water-based gels (hydrogels), such as colloidal silica, into a water saturated porous media is generally simulated under the assumption of miscible phases [26]. The injection of CS grout into a porous media can be simulated by coupling a transport equation with Darcy’s law and the continuity equation, as reported in the system of Eq. 1.

1 $$\left\{\begin{array}{l}\frac{\partial {\varepsilon}_{p}\rho }{\partial t}+ \nabla \cdot \left(\rho {\boldsymbol{u}}\right)= 0\\ \frac{\partial {{\boldsymbol{c}}}_{{\boldsymbol{C}}{\boldsymbol{S}}}}{\partial t}+\nabla \left(-{D}_{CS}\nabla {{\boldsymbol{c}}}_{{\boldsymbol{C}}{\boldsymbol{S}}}\right)+u\cdot \nabla {{\boldsymbol{c}}}_{{\boldsymbol{C}}{\boldsymbol{S}}}=0\\ \frac{\partial {{\boldsymbol{c}}}_{{\boldsymbol{j}}}}{\partial t}+\nabla \left(-{D}_{j}\nabla {{\boldsymbol{c}}}_{{\boldsymbol{j}}}\right)+u\cdot \nabla {{\boldsymbol{c}}}_{{\boldsymbol{j}}}=0\\ u= -\frac{K}{\mu ({t, c}_{CS},{c}_{j})}\left(\nabla p+\rho ({c}_{CS},{c}_{j})g\right)\end{array}\right.$$

where *u* [m/s] is Darcy velocity field, *c*_*CS*_ is the mass fraction [kg/kg] of the CS, *c*_*j*_ is the mole fraction [mol/mol] of the chemical species for j species present in the simulation (i.e. Na^+^ and K^+^) and *p* [Pa] the fluid pressure. Density, *ρ* [kg/m^3^] and dynamic viscosity, μ[Pa·s], are generally assumed to be constant parameters, however, in the case of grout injection where the grout is gelling, they are treated as functions of time and concentration. ${\varepsilon}_{p}$ is the porous media porosity and K the medium permeability [m^2^].

### Dynamic viscosity of colloidal silica grout

μ[Pa·s] is the dynamic viscosity of the fluid, which is defined as $\mu = \rho \nu$, where ν is the kinematic viscosity [m^2^·s]. The kinematic viscosity of the water phase will change depending on the colloidal silica concentration, the porewater chemistry and the time. Colloidal silica is a dispersion of nanometre silica particles, which can be destabilised via chemical alteration of the suspension (change in pH or electrolyte concentration), temperature, or silica concentration. Once destabilised, the dispersed particles tend to network together. As the particle network develops, the fluid viscosity increases sharply, and eventually, once a connected matrix of particles is created, a gel is formed [42]. The gel time (Fig. 1) is generally defined as the intersection between the time axis and the tangent line of the end of the viscosity curve.

Colloidal silica particle size, particle concentration, colloid’s pH, electrolyte concentration, cation valency and cation species are all factors that affect the gel time and the viscosity increase of the colloidal silica mixture.

In this model the grout gel time, ${t}_{gel}$, is defined according to Pedrotti, Wong [70], as the sum of two exponential terms, the former taking into consideration the effect of different pH and the latter the effect of the electrolyte concentration:

2 $${t}_{gel}={\mathrm{A}}_{1}\cdot \frac{1}{{S}_{S}}{\cdot e}^{\left(-pH\right)}+{\mathrm{A}}_{2}\cdot {e}^{\left(-{\mathrm{A}}_{3}{S}_{S}{c}_{eq}+{\mathrm{A}}_{4}\right)\cdot \left(pH-{pH}_{IEP}\right)}$$

*A*_*1*_, *A*_*2*_, *A*_*3*_ and *A*_*4*_ are parameters of the gel time model. S_*S*_ [m^−1^] is the total specific surface of the silica particles (Eq. 3), pH is the pH of the pore fluid (which was considered constant), *pH*_*IEP*_ is the pH at the isoelectric point of colloidal silica, which is a property of the material and *d* [m] is the nominal diameter of the colloidal silica, which is a property of the grout.

3 $${S}_{S}=\frac{6}{d}(1-(1-{c}_{CS}))\frac{{\rho}_{CS}}{{\rho}_{w}}$$

It is worth noting that in Eq. 2, the electrolyte concentration of the grout is expressed in terms of equivalent electrolyte concentration, ${c}_{eq}$ [mol/L], and defined (Eq. 4) as the sum of the concentration, ${c}_{j}$ [mol/L], of each species of cations, *j*, that is present in the grout mix and multiplied by the valency value of the cation, *v*_*j*_, and the ratio between the molar mass of cation, *M*j, and the molar mass of the Na, *M*_*Na*_, that is taken as a reference cation.

4 $${c}_{eq}=\sum_{j}{c}_{j}{v}_{j}\frac{{M}_{j}}{{M}_{Na}}$$

The dynamic viscosity,ν, is then defined as follows:

5 $$\left\{\begin{array}{l}for t \le {t}_{gel}: \mu = {\mu}_{0}\\ for t > {t}_{gel } : \mu ={\mu}_{0}+ \left(\frac{\Gamma }{{t}_{gel}}\right)\left(t-{t}_{gel}\right)\end{array}\right.$$

Time, *t*, and gel time, *t*_*gel*_, are expressed in minutes [min], and Γ is expressed in mPa s. Γ was found to be 11,991 according to Pedrotti, Wong [70].

### Grout density

Upon gelling colloidal silica does not expand or shrink [42]. Hence, the colloidal silica density is assumed to be constant during gelling and equal to the density of the fluid at a given time and location, *ρ* [kg/m^3^] which depends linearly on the concentrations of the silica colloids and of any dissolved cations (Eq. 6).

6 $$\rho = {\rho}_{w}+\left({\rho}_{CS}-{\rho}_{w}\right){c}_{CS}+\sum_{j=1}^{N}\left({\rho}_{j}-{\rho}_{w}\right){c}_{j}$$

*ρ*_w_ is the density of the water, *ρ*_CS_ the density of the grout and *ρ*_j_ the density of the density of the water having the maximum concentration, *c*_*j*_, of cation *j*.

### Diffusion and instability problems

When the diffusion component, $\nabla \left(-{D}_{CS}\nabla {{\boldsymbol{c}}}_{{\boldsymbol{C}}{\boldsymbol{S}}}\right)$**,** is much smaller than the advection component,${\boldsymbol{u}}\cdot \nabla {{\boldsymbol{c}}}_{{\boldsymbol{C}}{\boldsymbol{S}}}$**,** the numerical solution of Eq. 1 becomes unstable as the Peclet number becomes large. This condition is well known to produce numerical instability [7, 8, 41]. There are several methods to reduce instability, the simplest being to increase the diffusion coefficient to reduce the Peclet number [83]. Given the reduced length (few tens of centimetres) and relative short time (less than an hour) of injection of the experiments simulated in this paper, the length travelled by each species during injection was minimal. Hence, it was considered beyond the scope of this paper to investigate other and more advance strategies to numerically reduced the Peclet number. The diffusion tensor *D* [m^2^/s], was assumed to be isotropic and to take the same value for both the colloidal silica and the cation species. In the following simulations, the diffusion coefficient was increased to reduce numerical instability, where required. The effect of this increase on the accuracy of model predictions is discussed.

## Experimental methods

A set of three injection tank experiments with varying porous media and pore fluids was created to develop and validate the colloidal silica transport model. The aim of the experiments was twofold: to simulate grout permeation in shallow subsurface conditions, and thus at low injection pressure; to test the prediction capability of the model when different cations or clay lenses were introduced.

Each experiment was created with a different combination of porous media and initial pore fluid chemistry. Experiment 1 was prepared with a homogenous porous media and tap water as the pore fluid. Experiment 2 was prepared with a homogenous porous media and synthetic seawater as the pore fluid, and Experiment 3 was prepared with a heterogenous porous medium and tap water as the pore fluid. During each injection phase different monitoring systems were also adopted. In Table 1, a summary of each experimental layout, injection programme and monitoring system is reported.

**Table 1:** Injection programme

Experiment	Porous media	Pore water	Injected fluid	Monitoring
1	Sand	Tap water	Tracer	EC/camera
			CS	EC/camera
			CS grout	camera
2	Sand	Saline water	Tracer	camera
			CS	camera
			CS grout	camera
3	Sand + clay lenses	Tap water	Tracer	camera
			CS grout	camera/ERT

### Injection tank: materials and experimental setup

A Perspex tank of the size of 1300 mm by 400 mm by 100 mm was created at the Geomechanics laboratory at University of Strathclyde.

**Fig. 2:**
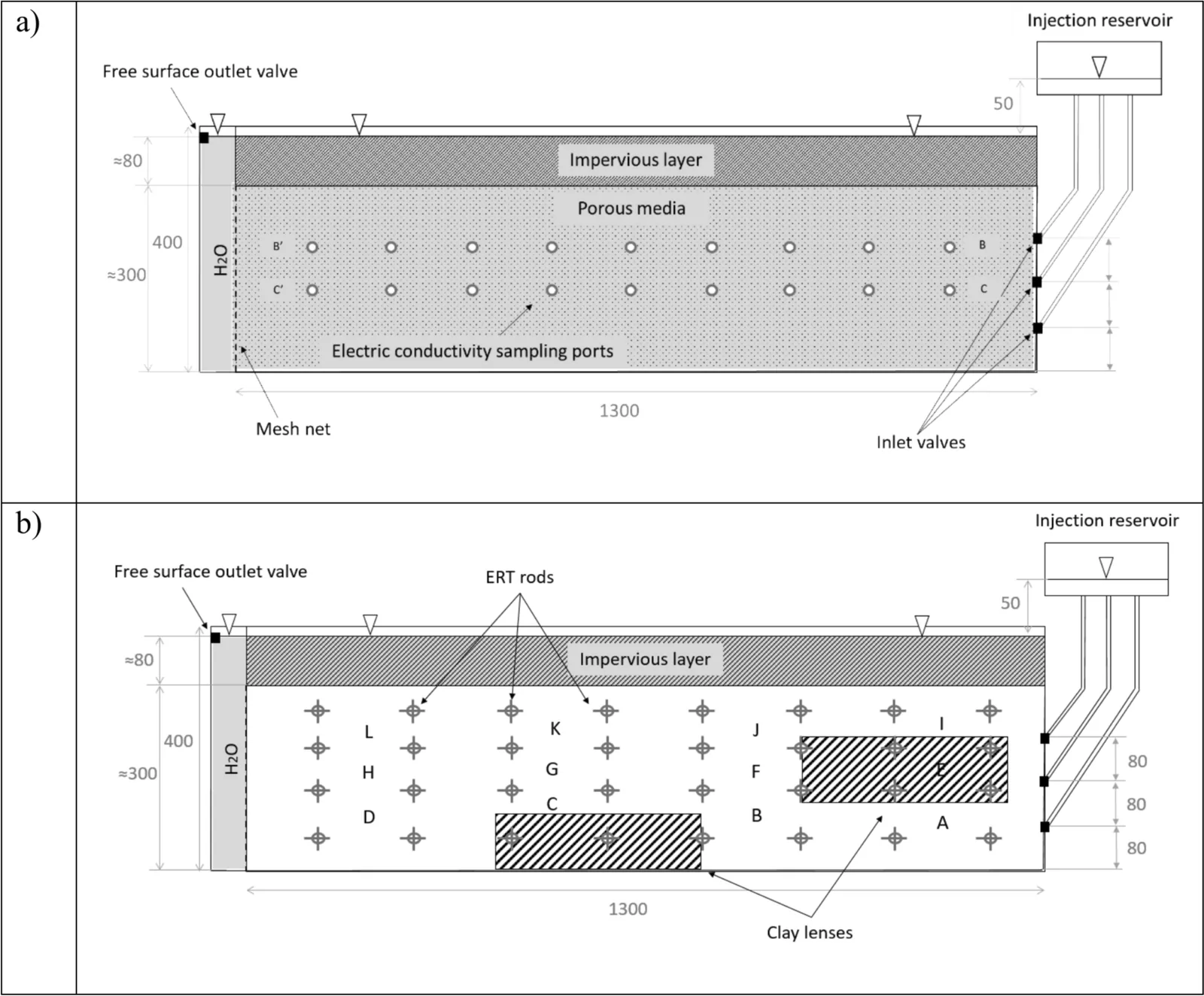
Experimental tank setup: **a** Homogeneous porous media and **b** Heterogeneous porous media

Figure 2 shows the two different tank configurations that were used. Injection into the tank was performed via three lateral valves connected to a constant-head reservoir on the right-hand side. On one side of the tank the three inlet valves were positioned at heights of 80, 160, 240 mm from the bottom. Each valve was 5 mm in diameter and was connected to a grout reservoir. To have a consistent energy loss, the three valves were connected to the reservoir by three flexible tubes of the same length, 1.5 m. Outflow was via a single outlet port on the opposite side of the tank from the injection valves.

For Experiments 1 and 2 (tank configuration shown in Fig. 2a) the tank was filled with approximately 72 kg of soil and 17 L of water. The porous media was created by water-pluviating Leighton Buzzard sand directly into the water-filled tank to guarantee full initial saturation of the sample. The sand has a median grain diameter d50 of 1.2 mm, specific gravity of 2.65 and coefficient of conformity of 1.26. This resulted in a final average porosity of 0.38. A layer of Speswhite kaolin clay was placed on top of the porous media to ensure horizontal flow by acting as a confined boundary (the impervious layer in Fig. 2). The clay was deposited on the top of the porous material in a slurry state, prepared at a water content equal to its liquid limit, taking care that it remained fully submerged during placement. In order to increase the consolidation state of the clay, drying and wetting cycles were performed. For the drying cycle the water level was lowered below the clay-porous media interface and then slowly raised. Care was taken to avoid crack formation during the drying stage. Since the clay layer was shrinking, additional clay was added during the consolidation process to guarantee good adhesion between the clay mass and the sides of the tank. Following these cycles, the water level was maintained to ensure the underlying sand remained completely saturated prior to the commencement of the grout injection. To avoid piping in the proximity of the injection points, a pressure of 10 kPa was applied to the first 30 cm of the impervious layer close to the injection point. Because Leighton Buzzard sand is a fast-draining material that is highly rigid in compression, and the applied + 5 cm injection head (~ 0.5 kPa) was an order of magnitude lower than the confining pressure, the sand matrix underwent negligible compression. The matrix remained mechanically stable throughout the injection phase, with no macroscopic deformation or alteration of the initial porosity visually observed. Pore fluid in Experiment 1 was created by using laboratory tap water equalised to a temperature of 18 °C (room temperature). A simplified synthetic seawater was prepared as the pore fluid in Experiment 2. The synthetic seawater was prepared by mixing 35 g/l NaCl to the laboratory tap water.

In Experiment 3 soil heterogeneity was created by placing two clay lenses within the sand. Figure 2b shows the alternate layout of the tank. The clay lenses were created by placing Speswhite kaolin (80% of grain size in the clays range) within homogeneous strata of Leighton Buzzard sand. The tank was then water saturated. As in the previous layout, a layer of Speswhite kaolin was place at the top of the tank, and consolidated, in order to create an impervious layer and force horizontal flow. As in Experiment 1, laboratory tap water was used as the initial pore fluid in Experiment 3.

### Injection experimental programme

As listed in Table 1, for each experiment, multiple injection phases were performed: a tracer; a colloidal silica suspension with no accelerant (“*CS*”); a grout mixture formed by a suspension of colloidal silica mixed with a saline accelerant (“*CS grout*”). The colloidal silica used was MP 320 part A, kindly donated by Master Builders Solutions. The physical properties of the colloid suspension are 40% silica mass concentration, a density of 1.3 kg/l and a nominal diameter of the silica particles of 15 nm.

Prior to the start of each experiment, the pore fluid was continuously injected in the tank until the electrical conductivity at the outlet achieved a constant value. This value was considered as the baseline. In each case, injection was performed using an injection head of + 5 cm. Under this constant pressure boundary condition, the theoretical cumulative injection volume is expected to increase proportionally to the square root of time. Rather than measuring volumetric flow directly at the inlet, the spatial progression of the grout front was monitored as a direct proxy for this injected volume. Water head at the outlet was kept constant and equal to the water level in the tank, at the top of the impervious layer (as shown in Fig. 2). To guarantee that the same initial conditions were applied to each injection phase, additional pore water was flushed through the tank between phases until the same electric conductivity baseline value was achieved at the outlet.

The tracer was prepared by hand-mixing pore water with 3 g/L of Fluorescein sodium (green dye). This enabled visualisation of the injected fluid front. The colloidal silica suspension with no accelerant (CS) was prepared by diluting the colloid suspension with tap water at a ratio of 5:1 (colloidal silica:water ratio) to replicate the silica concentration in the grout fluid (without cations present, the CS does not gel). No injection of CS without accelerant was performed in Experiment 3, to avoid CS trapping within the clay lenses.

Different CS grout mixtures were used for the three experiments. For each CS grout mixture, the amount of the electrolyte to be added to the CS solution and the concentration of each cations species was designed according to the gel time model presented in Pedrotti, Wong [70] and reported in Eq. 2.

For the CS grout mixture in Experiment 1, the colloidal silica was diluted using an electrolyte solution containing 1.7 M of NaCl, with a 5:1 hydrogel:electrolyte solution ratio, which resulted in a gel time of approximately 53 min after mixing. Gel time was checked ex-situ, prior to injection by means of a viscosity test performed on a small sample. For Experiment 1, CS grout injection started 30 min after grout mixing, so gelling was expected to occur after 23 min of injection.

To prove the capability of dealing with a grout mixture of different cations species, CS grout mixture in Experiment 2 was designed by using two different cations species: Na^+^ and K^+^. As in Experiment 1, the grout mixture was prepared with a 5:1 hydrogel:electrolyte solution ratio. However, to simulate an in-situ use of water for grout mixing, when near a coastal region, the electrolyte was prepared starting from the same synthetic seawater used as the initial pore fluid. In order to achieve the same gel time than Experiment 1 (i.e. 53 min), 9.317 g/L of KCl were added (calculation made according to the above-mentioned gel time model).

**Fig. 3:**
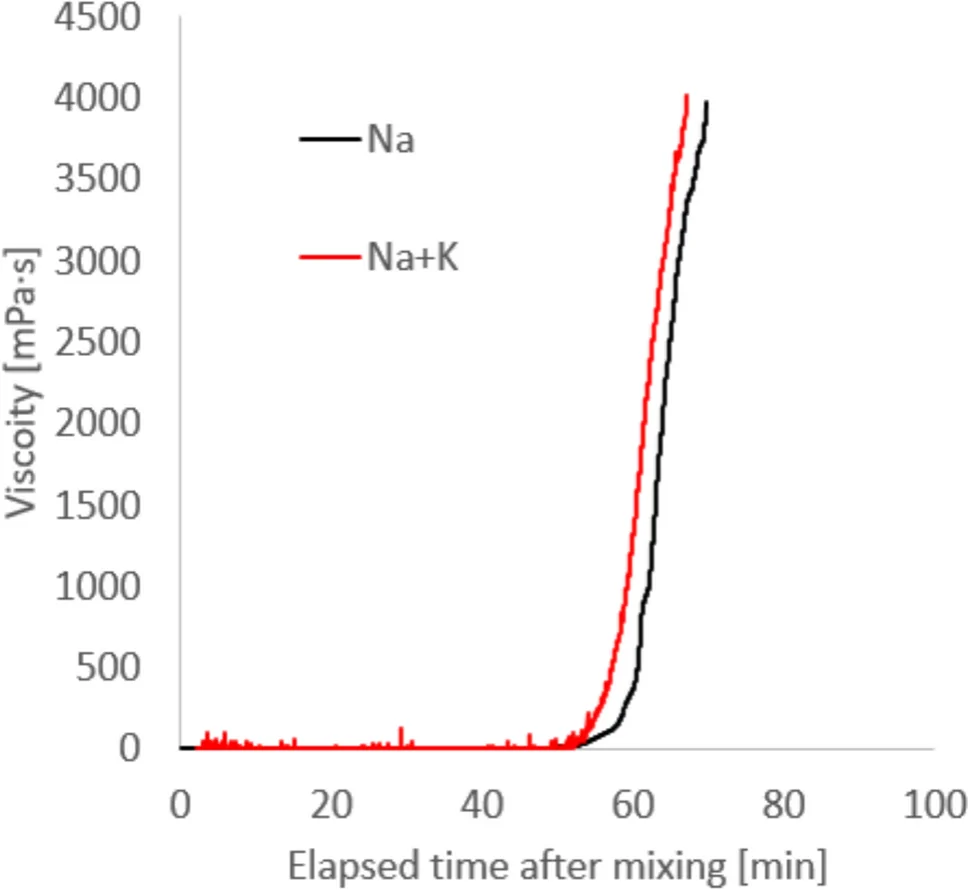
Viscosity curves comparison. Viscosity curve of the grout prepared with a NaCl electrolyte and viscosity curve of the grout prepared with an electrolyte mixture of NaCl and KCl

For the sake of completeness, Fig. 3 shows the two viscosity curves of CS grout used in Experiment 1 (CS grout prepared with a NaCl electrolyte) and Experiment 2 (CS grout prepared with a combination of NaCl and KCl electrolyte).

The colloidal silica grout in Experiment 3 was designed to have a gel time of 49 min, created by adopting a 4:1 colloidal silica electrolyte ratio and a NaCl concentration of 1.4 M. In Experiment 3, grouting commenced 15 min after CS grout mixing, hence the gel time was 34 min after injection commenced.

In each experiment, fluorescein was added to both CS and CS grout injection fluids with a concentration of 0.3 g/L. Preliminary tests confirmed that the addition of the fluorescein dye did not affect grout gelling in either injection fluid.

### Injection monitoring

All the performed injections were recorded with a Camera that took a digital image of the front side of the tank every 10 s. The resolution of each photo was 2048 by 1362 pixels.

During tracer injection in Experiment 1, pore water samples were collected from the 9 sampling ports in row CC’ and the 9 sampling port in row BB’ (Fig. 2) at 19 and 26 min, respectively. From each extraction point approximately 20 cl of liquid was collected. Since the electric conductivities prior to injection of the both the injected fluid and the pore fluid were known, this allowed the cation concentration at each sampling port to be calculated, via linear regression.

A time-lapse Electrical Resistivity Tomography (ERT) system was used to monitor the CS grout injection in Experiment 3. To test the concept of using ERT, preliminary ex-situ measurements of resistivity were taken on small samples of sand saturated with both the colloidal silica suspension (without the accelerator) and with the CS grout. Results indicated that the effect of the CS particles alone on resistivity was negligible, but that the cations in the accelerator solution caused a large change in resistivity: the resistivity dropped from > 150 Ω in water-saturated sand to a value below 15 Ω for CS grouted sand. Based on the promise of these data, an array of 32 electrodes was built in-situ for Experiment 3 by the British Geological Survey (BGS) to determine whether grout penetration could be detected in situ using ERT. The electrodes were inserted via the same sampling ports previously used to monitor the Electric Conductivity (EC) on the back of the tank (ERT rods in Fig. 2b). The ERT survey equipment was the same as that described in Kuras, Pritchard [48], which uses switched DC source signals with self-adapting current settings (input currents between 4 and 112 mA). Arrays were used in a dipole–dipole fashion. Data acquisition from the array was conducted every 24 s. The three-dimensional inversion was performed with the commercial code Res3DInvx64 [54] by assuming a rectangular grid.

### Day-after excavation

About 24 h after the end of each performed injection, the ungrouted mass of soil present in the tank was excavated with the aid of a shovel. To prevent any damage of the grouted surface, the ungrouted sand close to the grouted mass was removed by means of a commercial wet hoover (Henry Wet & Dry Corded Vacuum Cleaner). In this way, the success of grouting could be directly inspected, and the final shape of the grouted mass could be better highlighted.

## Numerical method

### The tank model set-up

Comsol Multiphysics® was used to implement the transport model in 3-dimensions. Equations (1) were solved to simulate tracer injection assuming a constant fluid density and viscosity. Equations (6) and (5), the density and viscosity equations respectively, were implemented to simulate injection of the CS and the CS grout. Neumann boundary conditions were imposed to describe the no-flow boundaries at the tank base and at the clay-porous media boundary at the top. The injection and outlet surfaces on the left and right-hand boundaries were assigned Dirichlet boundary conditions, where both the hydraulic head and the grout concentration were defined. The geometry of the model was as shown in Fig. 2. A mesh with tetrahedral elements (maximum and minimum size respectively 18.8 mm and 5.64 mm) was created, resulting in a total of 118,856 elements in the model. A MUMPS (MUltifrontal Massively Parallel sparse direct Solver) was applied and, in order to minimise the memory use, a segregated approach was chosen (the segregated variables were the species concentration and the fluid pressure). Table 2 summarises the input parameters for the tank simulation.

**Table 2:** Input parameters

	Parameter	Symbol	Experiment 1	Experiment 2	Experiment 3
Fluid characterisation	Pore water density	${\rho}_{w}$	1000 kg/m^3^	1035 kg/m^3^	1000 kg/m^3^
	CS density	${\rho}_{CS}$	1300 kg/m^3^	1300 kg/m^3^	1300 kg/m^3^
	NaCl molecular weight		58.88 g/mol	58.88 g/mol	58.88 g/mol
	NaCl concentration in the accelerator	${c}_{Na}$	1.7 M	0.59 M	1.4 M
	KCl molecular weight		74.55 g/ml	74.55 g/ml	74.55 g/ml
	KCl concentration in the accelerator	${c}_{K}$	–	1.4 M	–
	Silica/Grout ratio		5/6	5/6	4/5
	Water viscosity	${\mu}_{w}$	0.001 Pa*s	0.001 Pa*s	0.001 Pa*s
	CS viscosity	${\mu}_{CS}$	0.005 Pa*s	0.005 Pa*s	0.005 Pa*s
Calibration	Diffusion coefficient	D	0.01 m^2^/s	0.01 m^2^/s	0.01 m^2^/s
	Hydraulic permeability	k	1.89e-9 m^2^	1.89e-9 m^2^	1.89e-9 m^2^
Gel time model		A1	0.6554	0.6554	0.6554
		A2	27.83	27.83	27.83
		A3	1.7762	1.7762	1.7762
		A4	1910.5	1910.5	1910.5
		pH	8.6	8.6	8.6
		pH_IEP_	2	2	2
	Silica particle diameter	d	16 nm	13.9 nm	15.8 nm
Bezier	Bezier weight	w_1_	1	1	1
	Bezier weight	w_2_	0.85	0.85	0.85
	Bezier weight	w_3_	0.11	0.11	0.11
Spline	Spline viscosity slope	Γ	11,991 mPa*s	11,991 mPa*s	11,991 mPa*s

### Tank model calibration

The model parameters (diffusion coefficient *D* and hydraulic conductivity *k*)s were calibrated against the experimental data collected during tracer injection in Experiment 1.

**Fig. 4:**
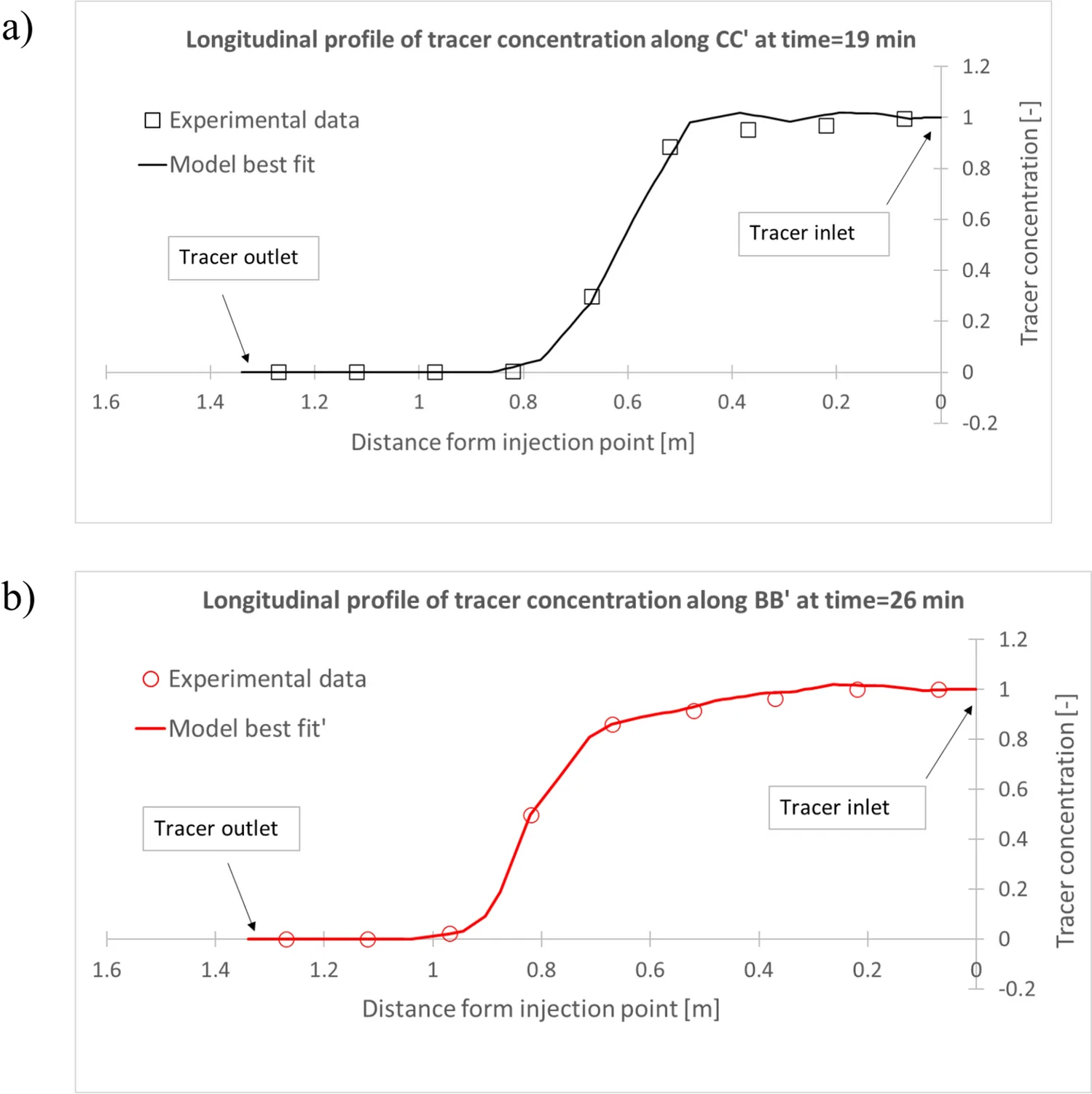
Parameter calibration: **a** Longitudinal profile of tracer concentration at 19′ and best fit simulation and **b** Longitudinal profile of tracer concentration at 26′ and best fit simulation

Figure 4a and b show the two horizontal profiles of tracer concentration derived from the EC measurements at the sampling ports in Experiment 1 at 19 min (row C, Fig. 2) and 26 min (row B, Fig. 2) respectively. The tracer injection was conducted twice to check for repeatability, and the experimental results were found to be almost identical, with a mean difference in tracer concentration of 0.018. Hence, only one set of tracer experiments is presented in Fig. 4.

In Fig. 4, the numerical simulation results are shown alongside the experimental data. The Root Mean Square Error (RMSE) of the calibration is 0.029 for the 19-min profile and 0.011 for the 26-min profile. The calibrated isotropic diffusion coefficient, *D,* is 0.01 [m^2^/s]. This value was selected as a compromise between achieving a good fit to the experimental data, whilst maintaining a relatively stable convergence of the solution (only a small oscillation is visible in the model results in Fig. 4a). The calibrated sand permeability was 1.$89\cdot 1{0}^{-9}{m}^{2}$. These values for the diffusion coefficient and the permeability were used in all three Experiments (Table 2). Tracer injections in Experiments 2 and 3 confirmed that the values remained a good fit to the data.

**Fig. 5:**
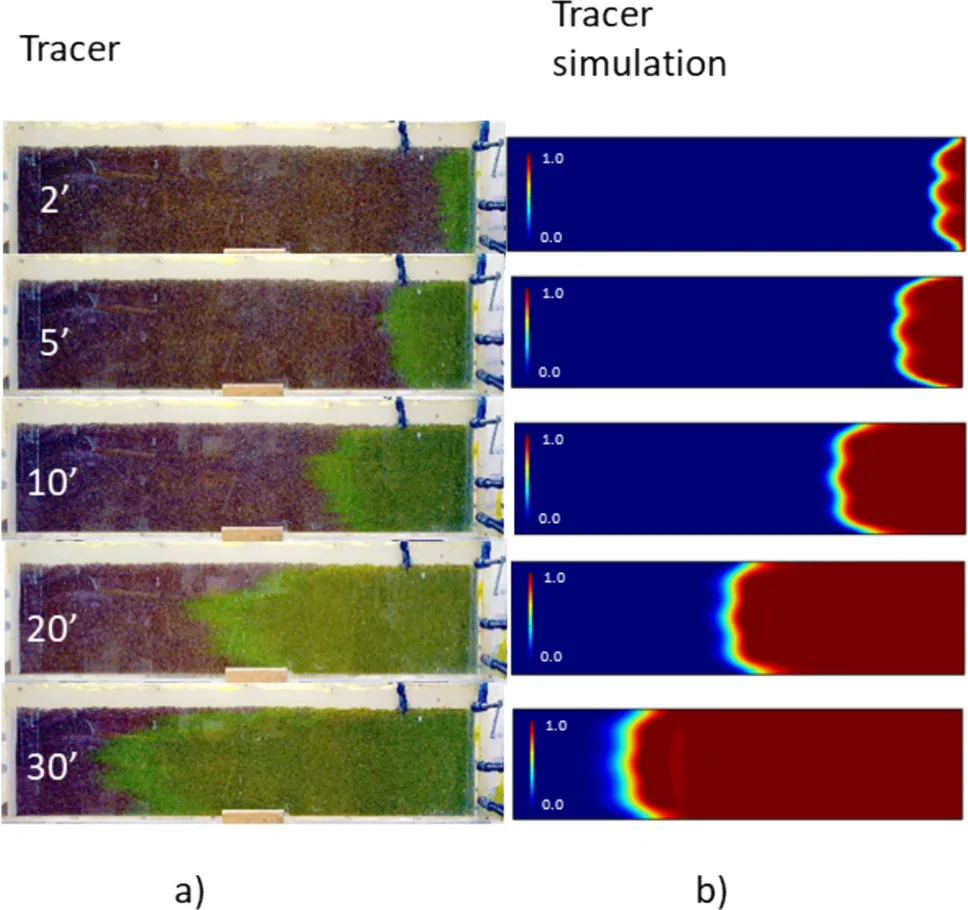
Experiment 1. Tracer injection: experimental (**a**) and numerical results (**b**)

Figure 5a shows a time-lapse of the injected fluorescein tracer (green) at 2, 5, 10, 20 and 30 min after injection for Experiment 1. Flow is from right to left in the tank and is relatively uniform and horizontal. Note that flow at the bottom and top of the sand-filled tank is slower due to frictional boundary effects adjacent to the Perspex base and the impervious layer, respectively. Figure 5b shows the model simulations of tracer injection using the values of the diffusion coefficient and the permeability that were calibrated from the data in Fig. 4. Some instability in the numerical solution is visible from the oscillations in the predicted contaminant front. Interestingly, the instability produces a similar behaviour to the ‘fingering’ that is visible in the experiment at the dye front, which is likely caused by small-scale variations in the packing density of the sand within the tank.

## Experiment 1—injection into a homogeneous sand

To test the capability of the model, two subsequent injections were conducted in Experiment 1. First, the CS suspension only was injected (without the accelerant) to determine the model’s capability to simulate penetration of the CS suspension; the CS is slightly denser than the in situ porewater and hence transport will differ from the fluoresceine tracer. Second, the CS grout was injected to explore the capability of the model to accurately predict grout final penetration and gelling.

### Injection of colloidal silica suspension—validation of the model with fluids of variable density

**Fig. 6:**
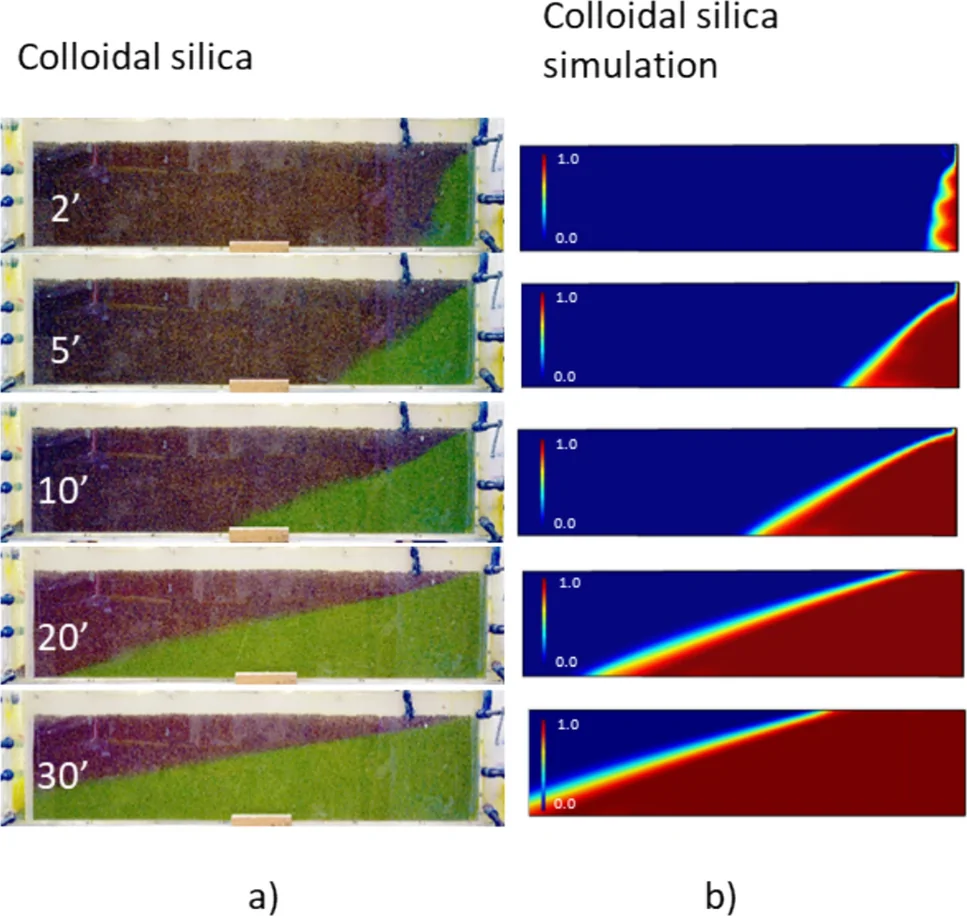
Experiment1. Colloidal silica injection: experimental (**a**) and numerical results (**b**)

The injection of CS (no accelerant added) into the tank allows for model validation in the case of injecting a miscible fluid with a different density to that in the porewater. Figure 6a shows a time-lapse of the CS injection. The effect of the higher density is immediately apparent; the concentration front is no longer vertical, the CS density is higher than that in the porewater, so it sinks towards the base of the tank whilst still moving from right-to-left in the image. Comparing the injection of the tracer (Fig. 5a) with the injection of CS (Fig. 6a) for a given time, horizontal CS transport is slower at the top part of the tank and faster at the base. Figure 6b shows the model predictions, the change in the shape of the front due to the increased density of CS is well predicted. Numerical instabilities at the front that were present in the tracer simulation, are no longer visible. This is probably due to the increase in Peclet number in the CS.

### Grout injection

**Fig. 7:**
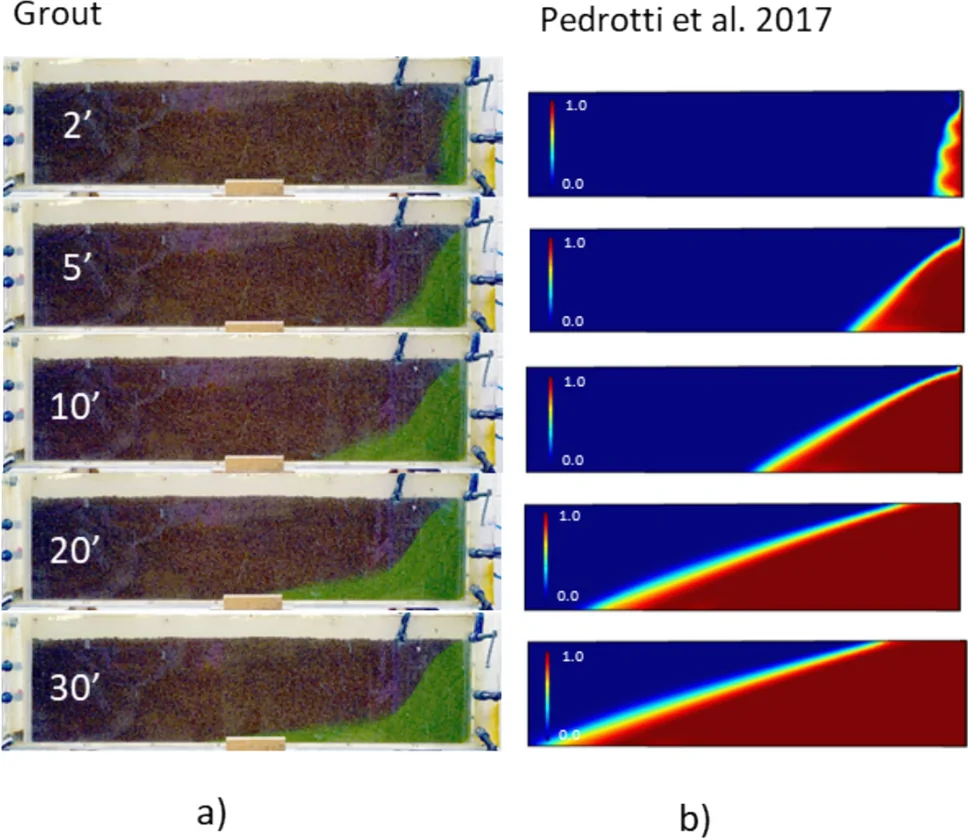
Experiment 1. **a** Grout injection and **b** grout simulation based on viscosity trend according to Pedrotti et al. [70]

Figure 7 shows a time-lapse of the experimental CS grout injection, alongside results from the grout model simulation. Immediately apparent in the experiment (Fig. 7a) is that after only 5 min of injection the grout has a lower flow rate than expected when compared to the model simulation (Fig. 7b) and also to the CS only experiment in Fig. 6a. After 10 min of injection (i.e. 40 min after grout mixing) no further grout permeation occurs, and the grout front remains stationary. Not only is the horizontal grout permeation far less than expected, so is the vertical permeation; the grout does not sink as rapidly as expected, producing a more vertical profile to the grout-front. As described in Sect. 4, the grout was expected to gel 23 min after the injection began, however, penetration into the sand stopped after only 10 min.

Grout gel time and the change in grout viscosity were modelled according to the analytical equations presented in Pedrotti, Wong [70] where dynamic viscosity is assumed to follow a bilinear Spline trend over time, as described in Eq. 5. Viscosity is, therefore, assumed to be constant until time is equal to the gel time (i.e. for the first 23 min of injection) and then to increase linearly. Clearly these assumptions are incorrect, since grout transport stops within 10 min of injection (Fig. 7a) suggesting that the grout viscosity increases much earlier.

### Improving the grout viscosity model: validation of the updated grout model

An improved grout viscosity model, that better fits the viscosity curve immediately prior to gelling, was developed using a 3-point Bezier weighted curve. A Bezier curve is a smooth continuous curve that can be used to approximate any real-world shape, and is commonly applied in computer graphics [102]. It requires the definition of an initial point, an end point and an additional point which control the smoothness of the curve. To individuate the 3 reference points to be used, gel time was used to nondimensionalise the viscosity curve, such that the non-dimensional gel time was equal to 1. For each nondimensionalised curve, the three chosen reference points were A(t_A_ = 0, μ_A_ = 0), B(t_B_ = 1, μ_B_ = 0) and C(t_C_ = 1, μ_C_= μ *), where *t*_*B*_ and *t*_*C*_ correspond to the nondiminesionalised gel time, and μ* is the viscosity at the gel time. μ* was statistically evaluated using a dataset of 51 different viscosity curves, as described in Appendix 1. It is worth noting that the choice of the 3 reference points is not crucial for fitting the viscosity curve, as long as one of them is linked to the gel time. Under the assumption that each viscosity curve will pass through the point defined as (*tgel, *μ*), it is possible to link the viscosity curve with the analytical model presented in Pedrotti [70] (and reported in the previous sections of this paper) and hence its dependency on the pore water-grout chemistry. In this way a bundle of curves passing through the critical point will be defined.

A quadratic form of a Bezier equation was applied [33]. In Eq. 7 the viscosity is expressed as a function of the parametric variable *h*, according to the general Bernstein-form.

7 $$\mu \left(h\right)= \sum_{i=0}^{n}\left(\left(\genfrac{}{}{0pt}{}{n}{i}\right){(1-h)}^{n-i}{h}^{i}{v}_{{P}_{i}}\right) 0\le h\le 1$$

where *P*_*i*_ are reference point having coordinates ${(t}_{{P}_{i}},{v}_{{P}_{i}})$. For three reference points on the curve (A, B and C), this reduces to:

8 $$\mu \left(h\right)={\left(1-h\right)}^{2}{v}_{A}+2\left(1-h\right)h{v}_{B}+{h}^{2}{v}_{C} 0\le h\le 1$$

If a weight is applied to each of the three reference points, the curve gains three degree of freedom (${w}_{A}$, ${w}_{B}$ and ${w}_{C}$) providing a better approximation of any arbitrary shape.

In Eq. 7, the dynamic viscosity is based on the parametric variable *h,* which is expressed as a function of the nondimensionalised time*, t*:

9 $$\begin{aligned} \mu \left( h \right) &= \frac{{\left( {1 - h} \right)^{2} v_{A} w_{A} + 2\left( {1 - h} \right)hv_{B} w_{B} + h^{2} v_{C} { }w_{C} { }}}{{\left( {1 - h} \right)^{2} w_{A} + 2\left( {1 - h} \right)hw_{B} + h^{2} w_{C} }}{ }0 \le h \le 1 \hfill \\ h\left( t \right) &= { }\frac{{t_{A} - t_{B} + \sqrt {t \cdot t_{A} - 2t \cdot t_{B} + t_{B}^{2} + t \cdot t_{B} - t_{A} \cdot t_{C} } }}{{t_{A} - 2t_{B} + t_{C} }} \hfill \\ \end{aligned}$$

where ${\mu}_{A}$, ${\mu}_{B}$, ${\mu}_{C}$ and ${t}_{A}$, ${t}_{B}$, ${t}_{C}$ are respectively the viscosity and time of the reference points A, B and C as previously defined. ${w}_{A}$, ${w}_{B}$ and ${w}_{C}$ are weights on the points.

Calibration of the resulting viscosity equation (Eq. 7) and comparison to a similar approach using an exponential curve (similar to what suggested by by Finsterle, Moridis [27]) is given in Appendix 1.

**Fig. 8:**
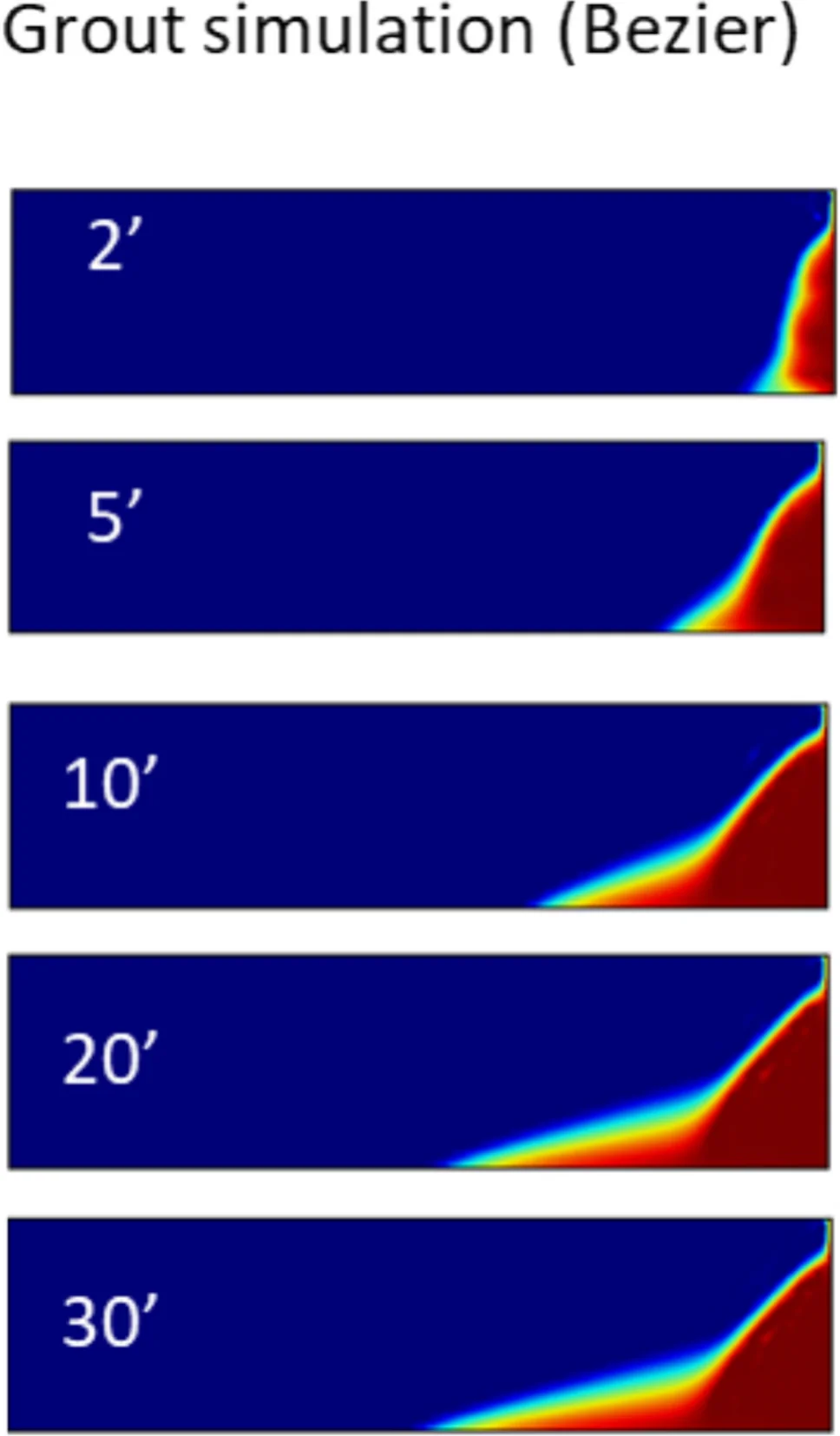
Experiment 1. Grout injection simulation with the updated viscosity model

The new viscosity equation (Eq. 7) was incorporated into the grout transport model and the simulation of Experiment 1 was repeated. Figure 8, shows the new model predictions. The location and shape of the grout front over time in the simulation is now a good match to the experimental data in Fig. 7a.

## Grout injection in a homogeneous media with saline pore-water

To test the capability of the model to predict colloidal silica grout injection into a porous material saturated with saline water, Experiment 2 was prepared with a simple synthetic seawater as the initial pore water. As described in Sect. 3.2, the CS grout was prepared using a mixture of Na^+^ and K^+^ cations as the accelerator.

### Experimental results and model simulation

**Fig. 9:**
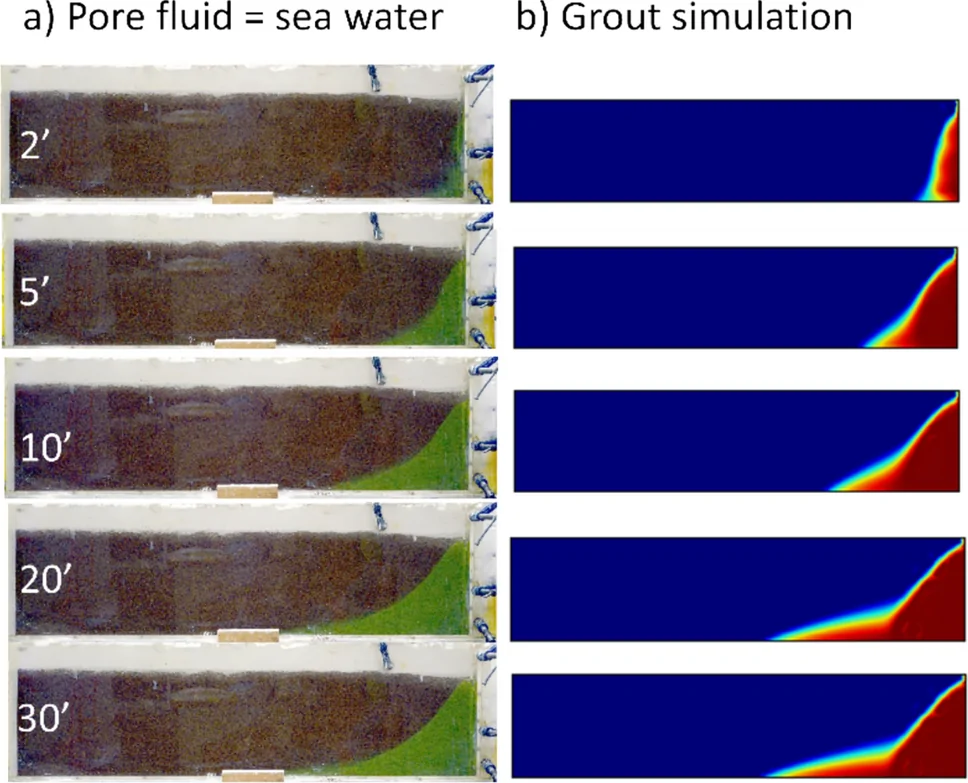
Experiment 2. Grout injection in synthetic seawater. **a** Experimental data and **b** simulation

In Fig. 9a grout injection into a seawater saturated tank is shown for 2, 5, 10, 20 and 30 min after the start of the injection. In Fig. 9b the simulation of the grout injection for Experiment 2 is shown. Despite the grout being prepared with two different cation species in the accelerator (K^+^ and Na^+^) and Na^+^ being present in the pore water, the model predicted grout front advancing correctly. Both in the experimental data and the simulation, the grout front appears sharper in Experiment 2 when compared to Experiment 1. Such effect is associated with a reduced diffusion potential between the grout and the seawater, and thus slower diffusion phenomena. In Experiment 2, the delta concentration between the grout and the pore water, $\nabla {c}_{i}$, that contributes to the diffusion term in Eq. 1, is smaller than in Experiment 1 for both cations species (i.e. Na^+^ and K^+^). In Experiment 1, the $\nabla {c}_{Na}$ is 0.283 M whereas in Experiment 2, $\nabla {c}_{Na}$ and $\nabla {c}_{K}$ are 0.000 and 0.233 M respectively.

**Fig. 10:**
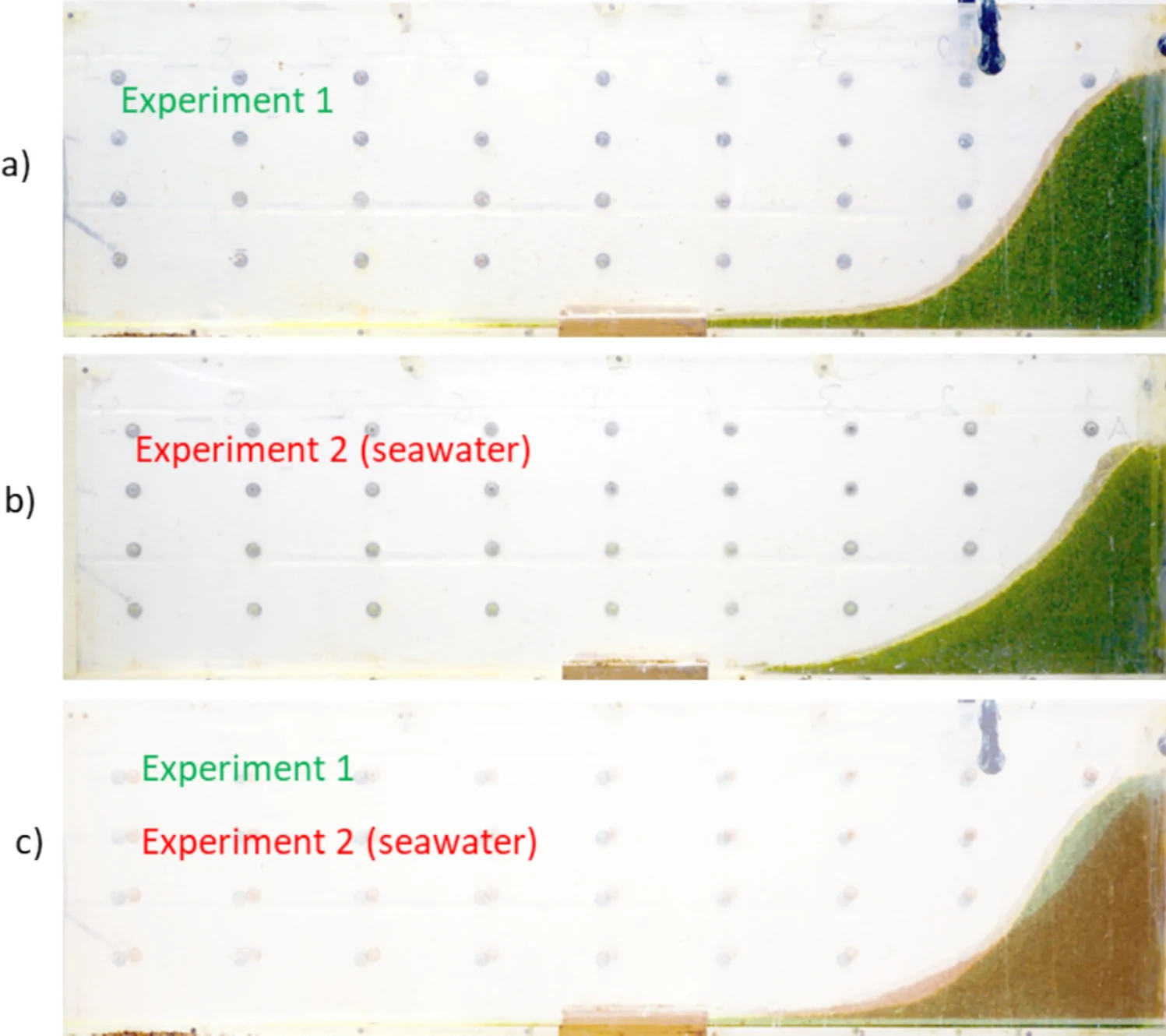
Day after injection grouted mass: **a** Experiment 1, **b** Experiment 2 (seawater) and **c** direct comparison

In Fig. 10, the final shape of the grouted masses for experiment 1 and 2 post excavation of the ungrouted sand is compared (Fig. 10a and b respectively). Although the shape of the two grouted masses is very similar, in the direct comparison in Fig. 10c it is visible that thanks to the smaller density difference between the pore water and the grout in experiment 2, the front of the grouted mass is much less vertical when compared with the one in experiment 1. In Experiment 2 the pore water is denser than in Experiment 1, in turn the pore water/grout density ratio is higher in Experiment 2 than Experiment 1 (0.935 and 0.909 respectively).

## Injection in media with clay lenses: experimental results, modelling and monitoring

Key to the uptake of colloidal silica grout in industry is the ability to perform engineering design calculations prior to a grouting campaign, and to demonstrate subsequent grout barrier integrity. Hence, in Experiment 3 grout penetration in the presence of discrete inclusions was investigated. Two clay lenses were placed within the sand. This experiment also provided a further platform for numerical model validation and the opportunity to test a potential field monitoring strategy; Electrical Resistivity Tomography (ERT) which has not previously been used to monitor colloidal silica grout.

### Grout injection and numerical simulation

**Fig. 11:**
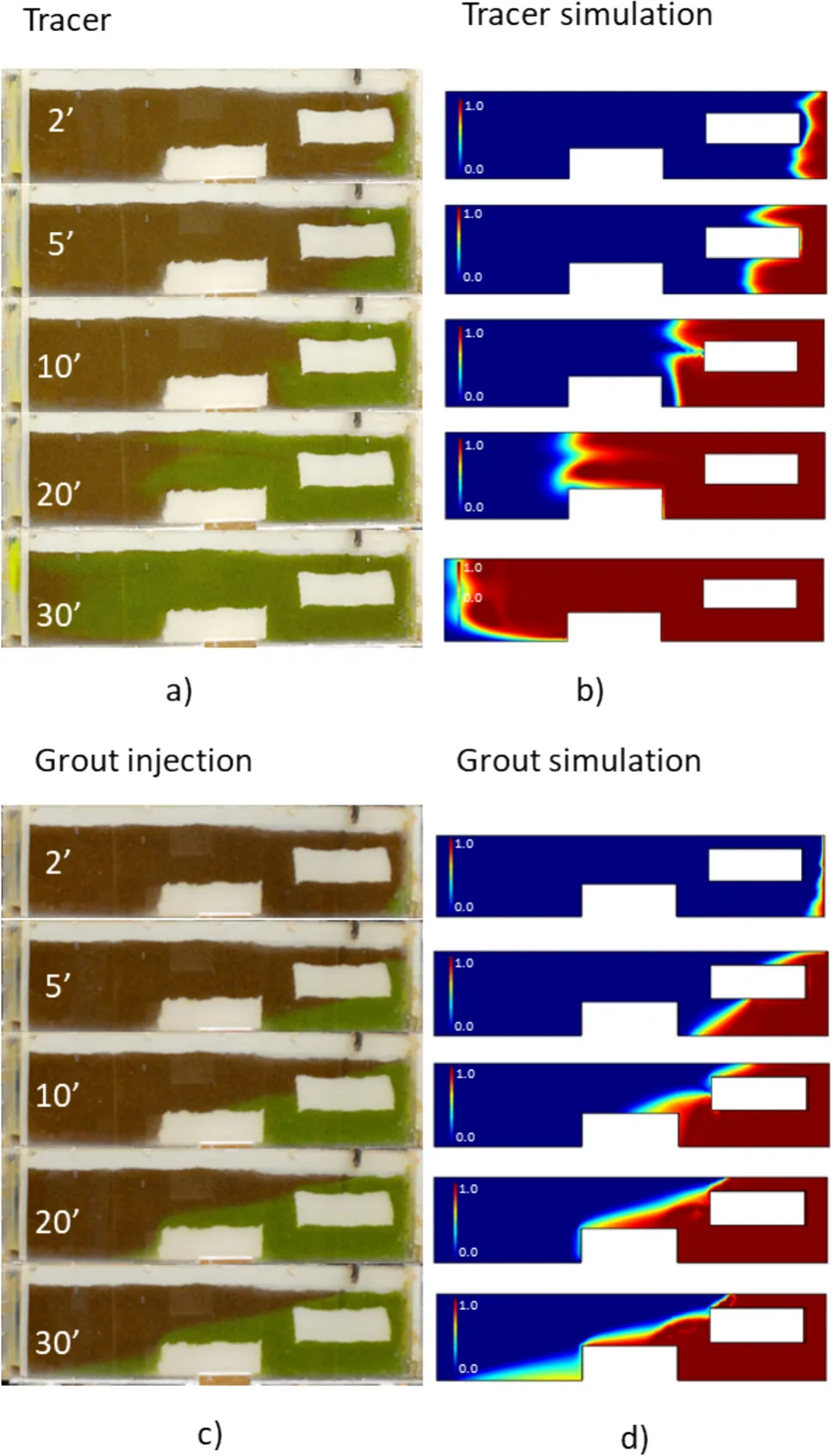
Experiment 3. Tank with clay lenses. Injection timelapse: **a** tracer injection, **b** tracer simulation, **c** grout injection, **d** grout simulation

Prior to grout injection, a tracer test was conducted to verify that the transport parameters derived from Experiment 1 were still valid and to determine the effect on tracer transport of the clay lenses. Figure 11a shows a time-lapse of the tracer experiment at 2, 5, 10, 20 and 30 min after injection started. Figure 11a shows that horizontal flow in the tank is disrupted by the presence of the two clay lenses, creating upward flow paths (t = 10 min) and channelling the flow between them. The difference in hydraulic conductivity between clay and sand is typically several orders of magnitude. Hence, during injection there is no visible penetration of the fluorescein dye into the clay lenses. The accompanying model simulation is shown in Fig. 11b, which demonstrates a good fit to the experimental data. To numerically simulate the discrete clay lenses present in Tank 3, these specific regions were defined as completely impervious domains (no-flow boundaries) within the active sand domain. This approach avoids the numerical instabilities associated with extreme hydraulic conductivity contrasts, while sufficiently replicating the complex, tortuous flow paths required to validate the grout’s rheological transport model.

Figure 11c shows the experimental results for injection of the CS grout. Once again, there is no visible penetration of the injected grout into the clay lenses. Also, despite the grout being denser than the pore fluid, the CS grout is channelled upwards (Fig. 11c, 10 min) between the two clay lenses. The grout flow rate gradually reduces, due to the viscosity increase, between 20 and 30 min after injection, finally stopping altogether after 24 min. Figure 11d shows the model simulation results for Experiment 3, the clay lenses in the model were considered as regions of zero permeability. The simulation accurately predicts the final shape of the grouted soil mass, as well as the evolving shape of the grout front over time.

### Grout monitoring using ERT

**Fig. 12:**
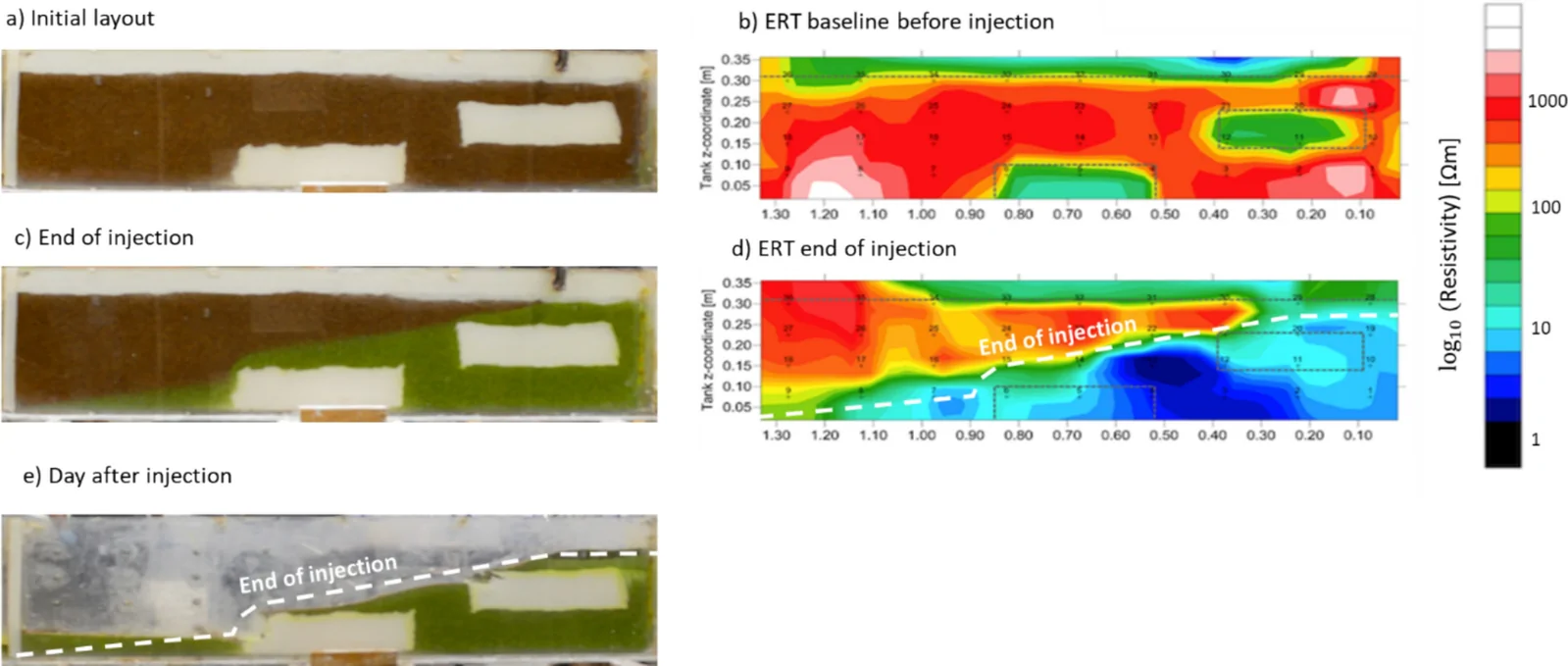
ERT monitoring. **a** Initial layout for tank with clay lenses, **b** ERT baseline, **c** camera image of the end of injection, **d** ERT tomography of the end of injection and **e** day after injection grouted mass

During Experiment 3, ERT monitoring was conducted. ERT scans of the tank were taken both prior to grouting, to determine the baseline resistivity, and immediately after grout injection was completed. Figure 12a and b show the initial fill material prior to grout injection alongside the baseline ERT scan. In the baseline ERT scan, the red areas have resistivities between 150 to 1500 Ω and clearly correspond to the saturated sand, whereas the green and blue areas have resistivities between 15 and 150 Ω and correspond to the saturated clay.

Figure 12c and d show the tank after grouting is complete, alongside the accompanying ERT scan. Red areas in Fig. 12d correspond to the higher resistivity regions of the ungrouted sand. Blue areas correspond to the low resistivity grouted sand and the clay lenses. The grout front is clearly visible and recorded by a sharp change in resistivity at the front (from blue to red in Fig. 12d and e confirms the shape of the grouted soil, the loose material was initially carefully excavated by hand, and then vacuumed to wash away any remaining loose particles. A comparison between the green grout front shown in Fig. 12c and the excavated grouted sand mass 1-day later (Fig. 12e) shows that some grout at the base of the tank on the left hand side has continued to gel overnight. This occurs because silica particles and accelerator diffuse into the porewater during injection at low concentrations at the grout front. Since this low concentration water is slightly denser that the initial porewater, it migrates downwards due to gravitational forces and gels at the base of the tank). For comparison, the digitised front from the photograph in Fig. 12c has been superimposed onto the resistivity map (white line in Fig. 12d) and onto Fig. 12e.

## Discussion

This study establishes that the permeation of colloidal silica (CS) grout can be accurately simulated within complex subsurface environments, even in the presence of saline porewaters. By refining the characterisation of the grout’s rheological evolution prior to gelation, this framework allows for the use of dilute accelerators to achieve extended induction periods—significantly exceeding the traditional one-hour window. From a geomechanical perspective, this is critical; extended gel times permit low-pressure injection, which mitigates the risk of hydraulic fracturing, ground heave, or structural disturbance to adjacent fragile nuclear infrastructure.

The coupling of a transport model with the analytical equation of the gel time presented in Pedrotti, Wong [70] provides the capability to predict the change in the grout properties (i.e. viscosity) based on the interaction with the cations present in the pore water. Since mixing phenomena at the grout front can be considered, it is possible to emplace the colloidal silica when different cations are already present in the ground. This could occur due to increased porewater salinity on coastal sites, or due to pre-existing ground contamination. Bots, Renshaw [13] discuss application of CS grout for in-situ lining of leaking legacy trenches where the surrounding soils already contain radioactive cations. They suggest that CS grout could be injected in situ to create a hydraulic barrier able to isolate the contaminated area from the surrounding water. In addition, they show the CS provides additional sorption capacity to enhance contaminant immobilisation. Our research would allow engineering design and post-emplacement verification of such a barrier.

A critical assumption of the proposed rheological model is that the colloidal silica grout behaves as a Newtonian fluid prior to gelation. This assumption is highly valid for the low-pressure permeation regime targeted in this study. As indicated by the experimental data shown above (Sect. 5.2 and Fig. 7),under low injection pressures, the driving force is insufficient to overcome increasing fluid resistance; consequently, flow arrests upon only a slight increase in viscosity, well before the nominal gel time is reached. Because flow ceases early in the viscosity evolution curve, fluid velocities and intra-pore shear rates remain strictly low. However, if this grout were deployed using high-pressure injection strategies, the fluid might exhibit non-Newtonian (shear-thinning) behaviour. In such scenarios, the proposed numerical framework would need to be coupled with a shear-dependent rheological model to maintain predictive accuracy.

Electrical Resistivity Tomography (ERT) serves as a vital bridge between numerical prediction and field reality. In field settings, where the baseline resistivity of natural soils might be highly variable or unknown, distinguishing between natural conductive clay lenses and the conductive saline grout using a single static ERT scan would be highly challenging. To overcome this, true differential (time-lapse) ERT analysis must be employed. This involves taking a baseline dataset prior to injection and mathematically inverting the difference between the baseline and subsequent monitoring datasets to isolate the dynamic resistivity changes caused by the advancing grout. While a side-by-side comparison of static inversions was sufficient for our visually controlled laboratory setup (Fig. 12), implementing mathematical time-lapse background subtraction is the critical strategy for isolating the grout signal in commercial applications. Furthermore, beyond simple post-injection detection, this differential ERT approach could provide quasi-live feedback on the progression of the grouted mass during the injection phase itself.

These features can be strategically integrated into the barrier design, either by ensuring they are encapsulated by the grout or by utilising their inherent low conductivity as part of the cut-off system. For example, a permeable layer could be deployed to form the base for a horizontal barrier. Subsequent phases of injection could be performed with decreasing depth, the presence of the pre-grouted layer would stop the grout from sinking and allow formation of a substantial horizontal barrier. In such a scenario, ERT can provide validation during the grouting campaign and inform on the required depths for subsequent injections.

## Conclusions

This paper presents a model able to predict the transport and gelling of colloidal silica grout in complex porous media. The primary novel findings from the numerical simulations demonstrate that: (1) dynamic changes in the grout's pre-gelling viscosity can be accurately predicted based on real-time mixing with varying cation concentrations in the porewater; (2) grout viscosity curves can be non-dimensionalised to produce a more accurate global relationship for viscosity evolution; and (3) colloidal silica permeation can be accurately simulated within highly heterogeneous subsurface environments, even in the presence of saline porewaters. The resulting viscosity relationship is implemented within a flow and transport model to predict grout permeation. By accurately predicting the rheology, the model validates the use of colloidal silica permeation under low pressure to mitigate the risk of ground heave and potential damage to neighbouring structures. The model predictions are validated using three experiments in which CS grout is injected into a m-scale tank with varying soil properties and groundwater cation chemistry. The model accurately predicts the location of the grout front in all three experiments. In the final experiment, the capability of ERT to image the grouted soil volume is explored. ERT accurately identifies the grouted soil volume using the change in resistivity between pre- and post-injection scans. This research opens the door to design and implementation of near-surface CS barriers for groundwater protection during decommissioning of legacy nuclear sites; the ability to model grout permeation with low injection pressures minimises the risk of ground heave and potential damage to neighbouring structures, whilst the post-injection ERT imaging capability provides assurance of barrier integrity and radionuclide containment.

## Figures and Tables

**Fig. 13:**
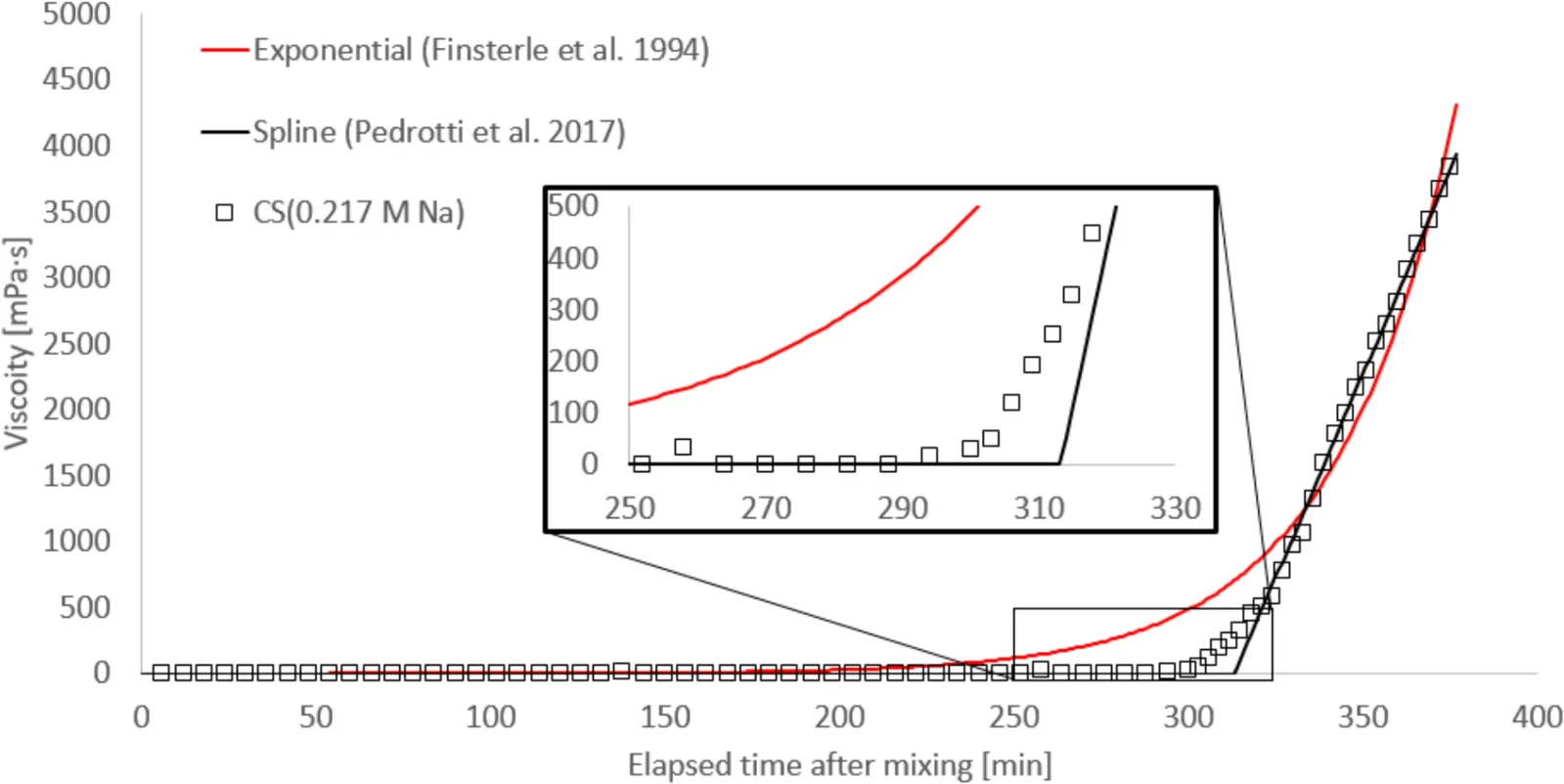
Fitting of the viscosity curve of the grout CS(0.217 M Na)

**Fig. 14:**
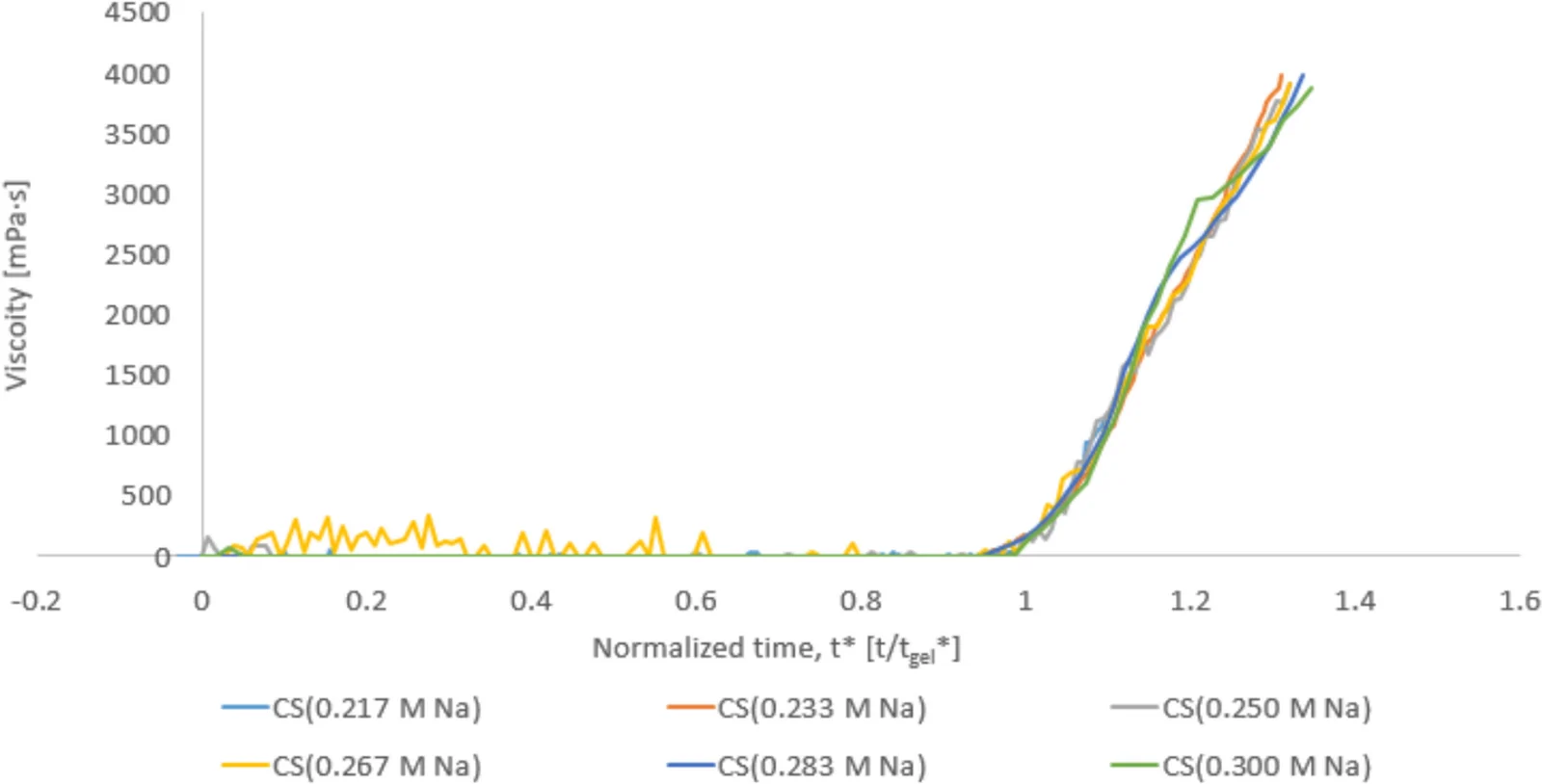
Time nondimensionalisation of the viscosity curves

**Fig. 15:**
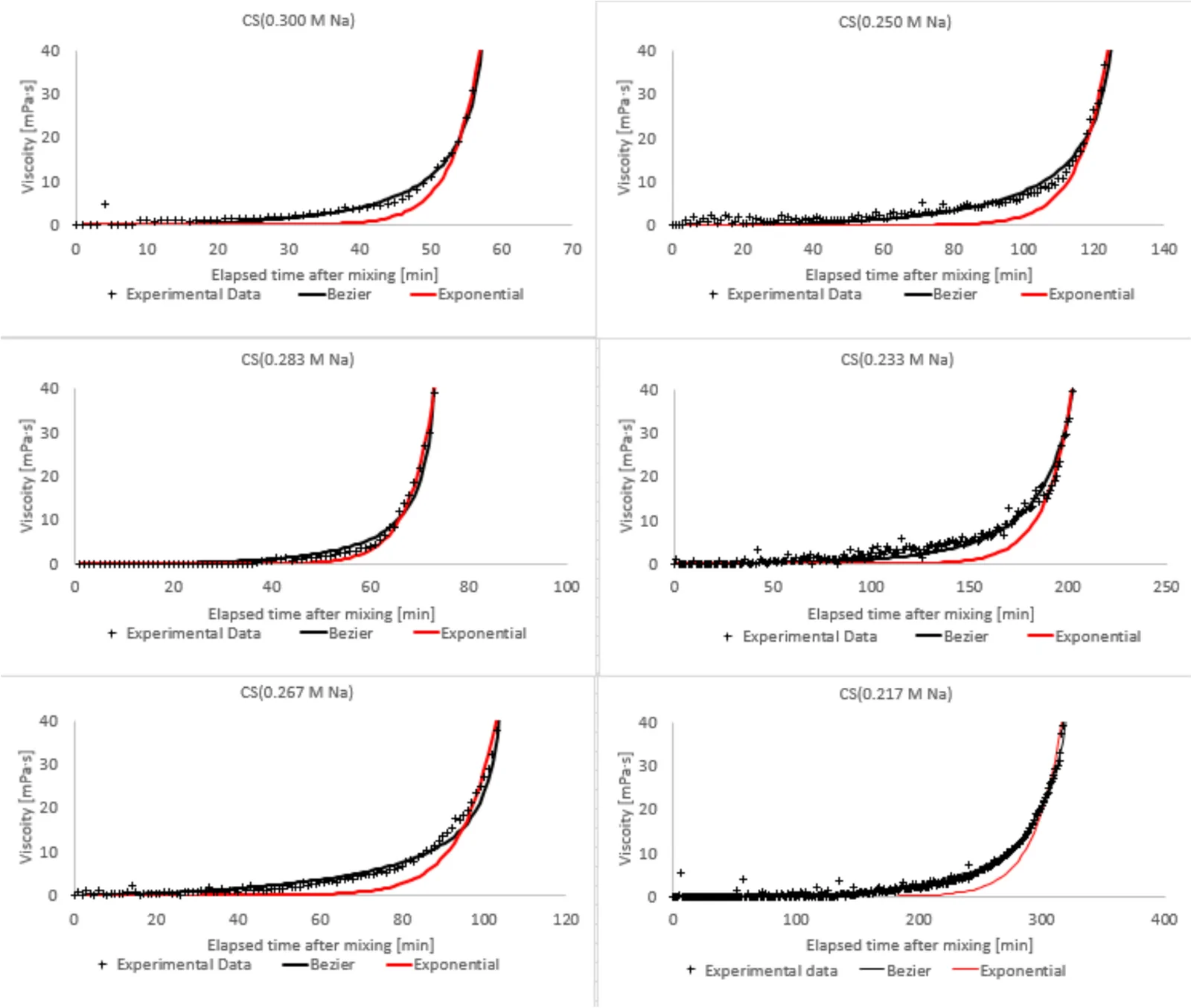
Viscosity curve fitting comparison

## Appendix 1: Modelling grout viscosity

Two viscosity models exist in the literature for colloidal silica grout: a two parameter bilinear spline (Pedrotti, Wong [70] which was summarised in Sect. 2.3 and a three parameter exponential curve initially presented by Finsterle, Moridis [27].

The three parameters of the exponential law were calibrated on the experimental measurement of the dynamic viscosity made on the grout before injection. This approach is more successful in capturing a reduction of the grout flow, however it underestimates the increase in viscosity occurring before the gel time, hence overestimates the grout flow. This results in an incorrect shape of the resulting grout front.

Figure 13 compares the viscosity curve of the grout injected in Experiment 1 with the best fitting model according to a two parameter bilinear spline (Pedrotti, Wong [70] and a three parameter exponential curve presented by Finsterle, Moridis [27]. The viscosity model propped in Pedrotti et al. 2017 provides the best-fit overall, but close to gelling it underestimates the measured grout viscosity.

To develop a more accurate model of grout viscosity over time, a dataset of 51 (https://github.com/PedroMat8/Pedrotti-et-al-2026-Acta-Geotechnica) viscosity curves was used. The time scale of each experimental viscosity curve was nondimensionalised using the gel time, *t*_*gel*_***, such that they all had a non-dimensional gel time equal to 1. For demonstration purposes the non-dimensional time, *t**, against the viscosity value, μ, for 6 of the 51 six viscosity curves is shown in Fig. 14.

From the whole dataset the viscosity value, μ*, corresponding to t* equal to 1, was calculated. In the calculation of the mean viscosity, 13 tests were considered as outliers in the normal distribution. This resulted in a mean viscosity of 138 mPa·s with a standard deviation of 36 mPa·s. From here on, it was assumed that any viscosity curve passes through the point (t = t_gel_, μ = μ *).

Similarly to what was described in Sect. 5.3 for the Bezier formulation, it is possible to link the viscosity curve with the analytical model presented in Pedrotti 2017 also for the exponential formulation presented by Finsterle [27](Eq. 10 and 11).

10 $$\left\{\begin{array}{l}\mu =A\cdot {e}^{Bt}+C\\ \widehat{\mu }= A\cdot {e}^{B{t}_{gel}}+C\end{array}\right.$$

This results in the following 2-parameter Exponential Model

11 $$\mu =A\cdot {(e}^{Bt}-{e}^{B{t}_{gel}})+\widehat{\mu }$$

To calibrate the two presented models, a set of viscosity tests were performed on 6 different grout mixes with NaCl concentrations of 0.217, 0.233, 0.25, 0.267, 0,283 and 0.3 M. For each viscosity test, 200 ml of grout was taken. Tests were performed with a Brookfield digital viscometer model LVT DV-II (spindle number 3 of the LV series). To achieve a good test resolution, tests were performed with a maximum recordable viscosity of 100 mPa·s, which guarantees a viscosity resolution of 10 mPa·s. At the beginning of each viscosity test temperature was 18 °C (± 0.5). During the viscosity test, the grout was covered to minimise evaporation.

A comparison of the optimal fit for both models, is shown in Fig. 15. The Bezier model provides the best fit to the experimental data in almost all cases. The three reference points used in the model were A(t_A_ = 0, μ_A_ = 0), B(t_B_ = t_gel_, μ_B_ = 0) and C(t_C_ = t_gel_, μ_C_= μ *) with optimal values for *w*_*A*_*, w*_*B*_ and *w*_*C*_ (Eq. 8 and 9) of 1,00, 0.85 and 0.11. It is important to note that the Bezier weights derived in this study represent empirical shape parameters defining a normalised single curve for the specific 40 wt% colloidal silica product tested. Because the data is non-dimensionalised against the gel time, *t*_*gel*_, these weights are completely independent of the varying porewater chemistry (e.g. cation concentration or ionic strength), which only acts to scale the temporal duration of the gelling process. However, for future applications utilising different base colloidal silica products, such as those with different particle size distributions or varying base silica concentrations, the shape of the non-dimensionalised viscosity curve may differ. In such cases, while the mathematical framework remains robust, the specific Bezier weights would need to be re-calibrated experimentally to capture the unique rheology of the new base product.
